# Multicolor and Warm White Emissions with a High Color Rendering Index in a Tb^3+^/Eu^3+^-Codoped Phosphor Ceramic Plate

**DOI:** 10.3390/ma12142240

**Published:** 2019-07-11

**Authors:** Haggeo Desirena, Jorge Molina-González, Octavio Meza, Priscilla Castillo, Juan Bujdud-Pérez

**Affiliations:** 1 Centro de Investigaciones en Óptica A.C., A.P. 1-948, León 37150, Mexico; 2 Instituto de Física, “Ing. Luis Rivera Terrazas’’, Benemérita Universidad Autónoma de Puebla, Apartado Postal J-48, Puebla 72570, Mexico

**Keywords:** Luminescence, Tb^3+^/Eu^3+^, white light, Luminous efficacy, high CRI, solid state lighting

## Abstract

A series of Tb^3+^/Eu^3+^-codoped phosphor ceramic plates with a high color rendering index (CRI) for a near-ultraviolet light emitting diode (LED) were fabricated. Color emission can be tuned from green to reddish as a function of Eu^3+^ concentration. By doping only 0.15 mol% of Eu^3+^ concentration, a comfortable warm white emission is promoted as a result of simultaneous emissions of Tb^3+^ and Eu^3+^ ions. A theoretical model is proposed to calculate the contributions of the emitted color of the donor (Tb^3+^) and acceptor (Eu^3+^) ions in terms of europium concentration. The energy transfer from Tb^3+^ to Eu^3+^ ions is corroborated by the luminescence spectra and decay time of Tb^3+^, with a maximum energy transfer efficiency of 76% for 28 mol% of Tb^3+^ and 14 mol% of Eu^3+^. Warm white LEDs were constructed using a 380 nm UV chip and showed a CRI of 82.5, which was one of highest values reported for Tb^3+^/Eu^3+^-codoped samples. Color-correlated temperature (CCT), color coordinate (CC), and luminous efficacy (LE) were utilized to know the potentials as a phosphor converter in solid-state lighting.

## 1. Introduction

In the last decade, phosphor-converted light emitting diodes (pcLEDs) have been introduced into the market to conquer the deficiencies of fluorescent and incandescent lamps. Current pcLEDs are basically a mixture of a phosphor converter and a silicon binder placed in the top of a blue LED chip (InGaN), and the combination of both colors yields white light. However, silicon binders that support the phosphor still present innate issues, such yellowing problems when exposed to a high density of energy caused by their low thermal conductivities [1]. In that sense, luminescence glasses, glass ceramics, phosphor in glass, and ceramic phosphor plates have emerged as good candidates to replace the silicon binder in high-power LEDs [2,3,4,5]. Among these approaches, special attention has concentrated on ceramic phosphor plates because of their superior thermal stability over different approaches for solid-state lighting [6]. It exhibits robustness, a homogenous luminescence emission, chemical stability, ageing resistance, and good thermal conductivity. The last property is key for the development of stable high-power LEDs and can be controlled through network formers, network modifiers, and network intermediators [7,8]. Even though this system shows attractive thermal and optical properties, most of the high-power systems using one single phosphor do not meet the characteristics for satisfactory color rendering index (CRI) and low color-correlated temperature (CCT) for residential lighting applications [9,10]. In that sense, a second phosphor to compensate the weak red emission of YAG:Ce^3+^ has been introduced, and the combination of both phosphors leads to warm white devices with low CCT and improved CRI [11]. 

Terbium (Tb^3+^) and europium (Eu^3+^) ions show prominent emission peaks in green and red regions, respectively. Such ions have been often used as phosphors in cathode ray tubes, plasma display panels, X-ray scintillation screens, and fluorescent lamps [12]. Recently, Eu^3+^-activated phosphors have been proposed as a new red emitter to compensate for the weak red emission of YAG:Ce^3+^-based pcLEDs. Results show that by choosing a proper host matrix, Eu^3+^ presents superior photometric properties compared to commercial CASN:Eu^2+^ phosphors under blue and UV excitation [13,14]. The narrow reabsorption of Eu^3+^ is the main advantage over nitride phosphors.

Tb^3+^/Eu^3+^-codoped materials such as glass, glass ceramic, and phosphor powder have been demonstrated to produce warm and cold white emission through energy transfer from Tb^3+^ to Eu^3+^ ions. Indeed, color emission and full conversion from green to red can be achieved by changing the ratio of the Tb^3+^ and Eu^3+^ concentration [15,16,17,18,19,20,21]. However, energy transfer efficiency and radiative decay probability depend strongly on the phonon energy of the host. Therefore, different promising host materials, such as oxides, oxysulfides, oxynitrides, chloride, and fluoride, have been investigated to develop efficient phosphors [22,23,24,25,26]. In that sense, special attention should be given to inorganic fluorides because of their low phonon energies that promote a decrease in the nonradiative decay probability. Presently, an NaYF_4_ fluoride crystal is one of the most efficient materials to produce upconversion emission as well as good chemical stability. However, tetragonal-phase LiYF_4_ crystals exceed the optical properties of NaYF_4_ because they promote stronger upconversion emissions and generate UV emission lines [27]. The crystalline phases of several LiYF_4_ samples have been synthetized by thermal decomposition, sol-gel, and Bridgman methods [28,29,30]. However, with these approaches it is impossible to sinter a phosphor plate. In that sense, a melt-quenching method shows greater potential than the methods mentioned above to synthetize phosphor plates with the desired shape and crystalline phase. These properties make LiYF_4_ an excellent host for Tb^3+^/Eu^3+^ ions to be used in solid-state lighting applications. 

In spite of several works that show that Tb^3+^/Eu^3+^-codoped materials are good candidates for solid-state lighting applications, most of the published reports only present spectroscopic and structural properties. However, luminous efficacy, lighting properties, and device prototypes based in Tb^3+^/Eu^3+^-codoped material for solid-state lighting have been seldom reported [31]. In this work, the color tuning of a Tb^3+^/Eu^3+^ ceramic phosphor plate as a function of Eu^3+^ concentration has been investigated. Color coordinate and color rendering indexes were measured to evaluate the potential of the phosphor converter in solid-state lighting.

## 2. Materials and Methods

Several LiY_1−x−y_F_4_:yTb^3+^/xEu^3+^ ceramic phosphor plates were prepared by a melt-quenching technique (where y = 28 mol% and x = 0.15, 0.3, 1.5, 8.5, and 14 mol%). In the composition, 5 mol% extra of TeO_2_ was used as a sintering additive to promote the formation of a ceramic plate. The total lanthanide concentration in the host matrix and other optical properties are show in Table 1. All reactants were 99.99% pure and used as received. The reactants were tellurium oxide (TeO_2_), lithium fluoride (LiF), yttrium fluoride (YF_3_), terbium fluoride (TbF_3_), and europium fluoride (EuF_3_). Calculated quantities of the chemicals were mixed in an agate mortar for 30 min and melted in a PDI electric furnace at 1100 °C for 1 h in alumina crucibles so that a homogeneously mixed melt was obtained. The reaction performance was 96%, indicating a low volatility of fluoride compounds. The samples were subsequently annealed at a temperature of 280 °C. The time to finish the annealing process took around 18 h. Samples were cut and then polished to 800 µm-thick slabs for different measurements. Photoluminescence characterizations were performed using a xenon lamp, monochromator 2300i from Acton Research (Trenton, NJ, USA) and R955 Hamamatsu photomultiplier tube (Hamamatsu, Japan). The decay profile (lifetime) corresponding to 545 nm and 613 nm was recorded using a pulsed UV LED (Opulent Americas, Raleigh, NC, USA) centered at 365 nm and a Teledyne LeCroy oscilloscope (HDO 5055, New York, NY, USA). Special care was taken to maintain the alignment of the set-up in order to compare the intensity of the visible signal between different characterized samples. The ceramic phosphor plate was placed on a commercial UV LED with an emission wavelength centered in 380 nm. An integrating sphere 1 m in diameter (Labsphere Co., North Sutton, NH, USA) was used to measure the CCT, CIE (Comission Internationale de l´Éclairage 1931) chromaticity diagram, color coordinates, and luminous efficacy (LE) with a bias current of 20 mA.

The crystalline structures of the samples were characterized using the X-ray diffraction (XRD) of Bruker instrument (D2 Phaser, Bruker, Billerica, MA, USA) equipment with Cu Kα radiation at 1.54184 Å. The recorded XRD diffractograms were obtained from 10 to 70° 2θ range with increments of 0.02° and a sweep time of 0.5 s. The SEM images were performed using a SEM of JEOL (JSM-7800F, Tokyo, Japan).

## 3. Results and Discussion

### 3.1. X-ray Diffraction (XRD) Characterization and Photoluminescence Properties

**Figure 1:**
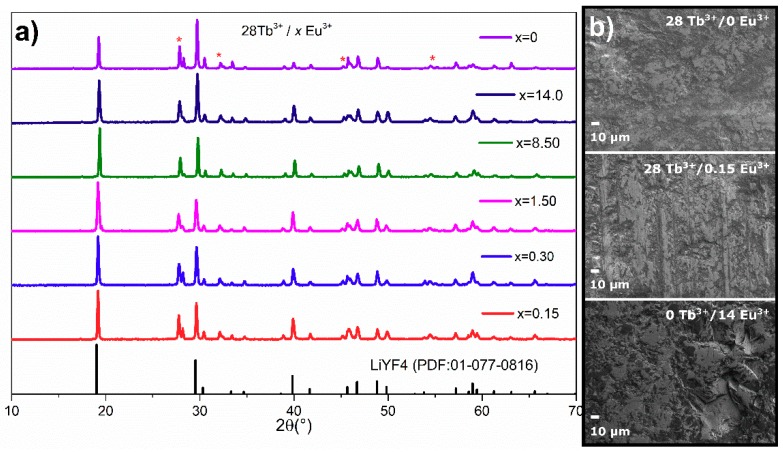
(**a**) X-ray diffraction pattern of the phosphor ceramic plate. The peaks marked with an asterisk correspond to the phase Y_2_Te_6_O_15_. (**b**) SEM image of the ceramic phosphor plate synthetized trough melt quenching method.

The composition and phase purity of Tb^3+^-doped and Tb^3+^/Eu^3+^-codoped samples with different rare earth concentrations were analyzed by XRD. As presented in Figure 1a, almost all diffraction peaks from the ceramic phosphor plate could be indexed to the standard tetragonal LiYF_4_ phase (PDF#77-0816) with low impurity phases, indicating that the samples were successfully crystallized, and TeO_2_ concentrations did not cause significant changes in the host structure. According to Figure 1a, it was confirmed that LiYF_4_ samples possessed a tetragonal crystal structure with space group I41/a. The obtained results were comparable to those reported by Kim et. al., where the tetragonal phase appeared as the main crystalline phase when the Eu^3+^ concentration was below 40 mol% in the LiYF_4_ matrix [28]. For this study, the total lanthanide concentrations in LiYF_4_ samples were 28, 28.15, 28.3, 29.5, 36.5, and 42 mol%. Additional impurity peaks appeared from 28 to 29.5 mol%, which were associated with a Y_2_Te_6_O_15_ (PDF#37-1393) phase, whereas from 36.5 to 42 mol% the peak centered at 2*θ* = 28.2° disappeared, and the purity of the tetragonal phase increased. The lanthanide concentration also modified the diffraction peaks, shifting to a high angle side, when the lanthanide increased from 28 mol% to 42 mol%. Such a fact was associated with the substitution of larger ionic radii of Tb^3+^ and Eu^3+^ by a smaller Y^3+^ ionic radius. An SEM image of 28 mol% of Tb^3+^, 28 mol% of Tb/0.15 mol% of Eu, and 14 mol% of Eu^3+^ are shown in Figure 1b. The increase in Eu^3+^ concentration did not promote a significant change on the surface of the phosphor plate.

**Figure 2:**
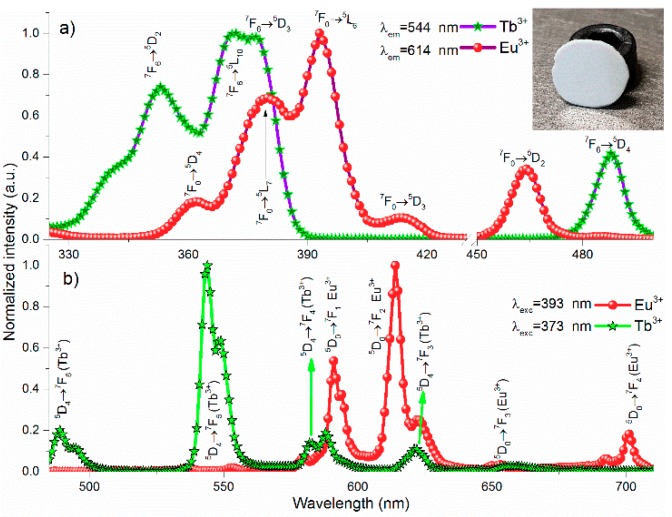
(**a**) Excitation and (**b**) emission spectra for Eu^3+^- and Tb^3+^-doped samples. The inset shows the picture of the opaque ceramic phosphor plate.

Figure 2a shows the photoluminescence excitation (PLE) from 325 to 500 nm in Tb^3+^- and Eu^3+^-doped ceramic plates monitored at 544 and 614 nm, respectively. The spectrum shows four main excitation bands centered at 353, 373, 378, and 485 nm, which were assigned to ^7^F_6_→^5^D_2_, ^7^F_6_→^5^L_10_, ^7^F_6_→^5^D_3_, and ^7^F_6_→^5^D_4_ transitions of Tb^3+^ respectively [29]. The ^7^F_6_→^5^D_3_ transition showed two excitation peaks at 373 and 378 nm, with the 378 nm shoulder being slightly weaker. From 331 to 338 nm there was a continuous excitation, while from 388 to 475 nm no excitation peaks were observed in Tb^3+^-doped samples. Figure 2b shows the photoluminescence emission (PL) of Tb^3+^-doped and Eu^3+^-doped ceramic phosphor plates under 373 and 396 nm excitation wavelengths. The bands centered at 490, 545, 589, and 622 nm were assigned to ^5^D_4_→^7^F_6_, ^5^D_4_→^7^F_5_, ^5^D_4_→^7^F_4_, and ^5^D_4_→^7^F_3_ transitions of Tb^3+^ respectively. These visible bands were the result of the well-known down-conversion process, and their emitted colors depended on the concentration of Tb^3+^ ions. In this work, the Tb^3+^ concentration was varied systematically (not show here) from 2 to 28 mol%, and results showed that there was no quenching concentration indicium. These results were similar to those found by other research groups, where 40 mol% of dopant ions were incorporated, and the green emission (545 nm) was the dominant color for the LiYF_4_ matrix. The inset in Figure 2a shows the picture of the opaque ceramic phosphor plate.

To distinguish the PLE spectra of Tb^3+^ and Eu^3+^, a 14 mol% of Eu^3+^-doped sample was synthesized. Figure 2a shows the excitation spectra of the Eu^3+^-doped ceramic phosphor plate recorded at 614 nm emission. The sample showed five dominant bands centered at 362, 380, 393, 414, and 464 nm, which were assigned to ^7^F_0_→^5^D_4_, ^7^F_0_→^5^L_7_, ^7^F_0_→^5^L_6_, ^7^F_0_→^5^D_3_, and ^7^F_0_→^5^D_2_ transitions of Eu^3+^ respectively. Upon excitation at 393 nm, a magenta color appeared, with a spectral range from 585 to 710 nm, which are shown in Figure 2b. The 592, 614, 653, and 701 nm bands were attributed to ^5^D_0_→^7^F_1_, ^5^D_0_→^7^F_2_, ^5^D_0_→^7^F_3_, and ^5^D_0_→^7^F_4_ transitions of Eu^3+^, respectively, where the 592 and 614 nm emission bands were the feature emissions for LiYF_4_:Eu^3+^. Among these transitions, the electric dipole ^5^D_0_→^7^F_2_ transition was the most intense, followed by less intense magnetic dipole ^5^D_0_→^7^F_1_ transitions. This indicated that Eu^3+^ ions were located at noninversion symmetric sites [32]. An attractive detail of the emission spectrum was observed at the 701 nm band, which typically was 40% less intense than the 614 nm band in the LiYF_4_ matrix [28]. However, in this work the 701 nm band was debilitated, as it was 78% weaker than 614 nm band. For solid-state lighting applications, the 701 nm band is a waste of energy because the eye sensitivity is zero, whereas 614 nm bands are considered the optimal red emission to obtain high luminous efficacy and high CRI in warm white LEDs [33]. 

**Figure 3:**
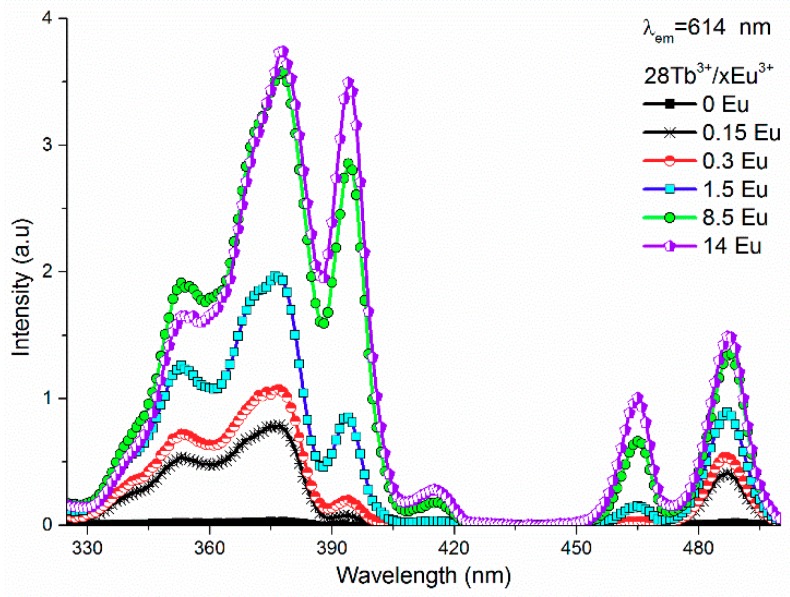
Excitation spectra of the Tb^3+^/Eu^3+^-codoped ceramic phosphor plate monitored at 614 nm.

Figure 3 shows the excitation spectra of the Tb^3+^/Eu^3+^-codoped ceramic phosphor plate. By adding only 0.15 mol% of Eu^3+^ to the Tb^3+^-doped sample, the intensity ratios between splitting peaks at 373 and 378 nm changed slightly, where the shoulder at 378 nm was more intense. This fact was due to the spectral overlapping of the excitation bands at 373 and 380 nm of Tb^3+^ and Eu^3+^ ions, respectively. Such bands became more pronounced when the Eu^3+^ concentration increased from 0.05 to 5 mol%. From the point of view of solid-state lighting, the red shift of the excitation bands became significant because the optical power in the commercial LED chip at 380 nm was higher than 365 nm, and there was a lower probability of dispersion at larger wavelengths. In addition, upon 378 nm excitation, both Tb^3+^ and Eu^3+^ were excited efficiently (vertical gray line), producing simultaneously green and red emission bands. As a result, a higher luminous flux was obtained from a phosphor converter device. As presented in Figure 2b, the excitation bands at 378, 393, 464, and 485 nm for Tb^3+^/Eu^3+^-codoped samples increased with the Eu^3+^ concentration; however, the intensity of the band at 393 nm depended strongly on Eu^3+^ content rather than the other bands.

**Figure 4:**
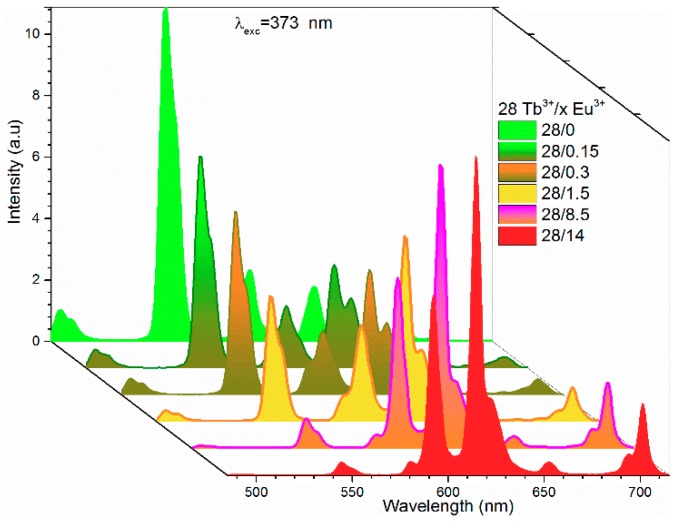
Emission spectra of the Tb^3+^/Eu^3+^-codoped ceramic phosphor plate excited at 378 nm.

Figure 4 shows the emission spectra of Tb^3+^/Eu^3+^-codoped samples as a function of Eu^3+^ concentration. Simultaneous emissions from Tb^3+^ and Eu^3+^ were observed under 378 nm, which indicated the existence of energy transfer between Tb^3+^ and Eu^3+^. Intensity of emission bands of Tb^3+^ at 490, 545, and 589 nm decreased monotonically as the Eu^3+^ concentration increased from 0.15 mol% to 14 mol%. Among these bands, the 545 nm emission was the most influenced band by Eu^3+^ content, which decreased 36% of the initial intensity with only 0.15 mol% of Eu^3+^. Concurrently, an increment of 33% was observed for the 614 nm emission band of Eu^3+^ ions. Figure 4 shows that almost all energy was transferred from Tb^3+^ to Eu^3+^ when the Eu^3+^ reached 14 mol%, where red was the main color.

### 3.2. Rate Equation Model and Energy Transfer

**Figure 5:**
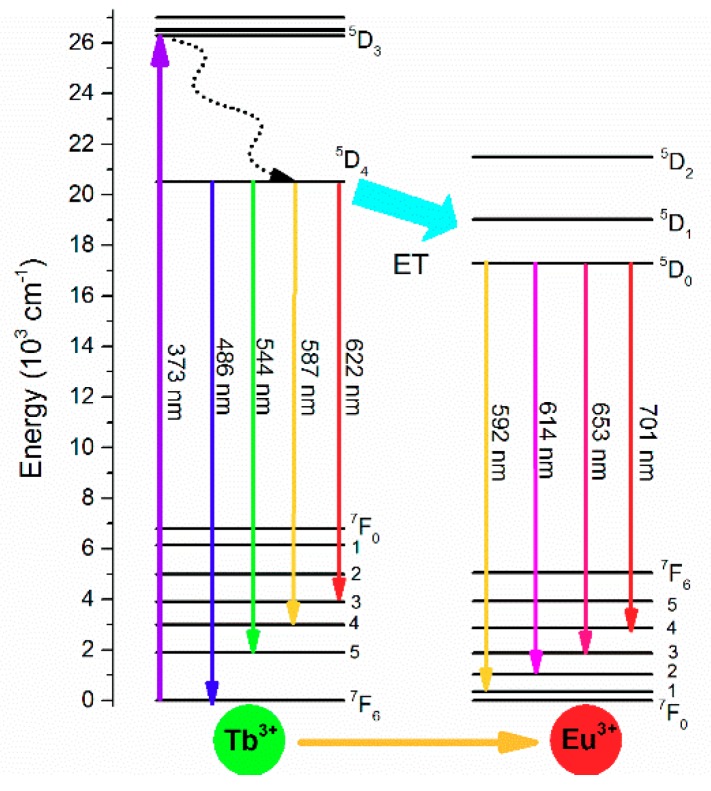
Schematic level diagram of Tb^3+^ and Eu^3+^ and the energy transfer mechanism.

To clarify the emissions corresponding to Tb^3+^ and Eu^3+^ ions, the following simplified model was proposed. (1) First, some Tb^3+^ ions were promoted from the ground state ^7^F_6_ to the excited ^5^D_3_ level as a result of pumping at 373 nm. The absorption rate was denoted by A_02_ (s^−1^). (2) Once some Terbium ions were in the ^5^D_3_ level, they relaxed nonradiatively to the ^5^D_4_ level; this multiphonon relaxation was denoted by A_21_ (s^−1^). Subsequently, two processes could occur, phonon relaxation A_10_ (s^−1^) or energy transfer from Tb^3+^ to Eu^3+^ ions (W). (3) Tb^3+^ emission wavelength peaks were 490, 545, 589, and 622 nm. (4) The energy transfer promotes some Eu^3+^ ions from the ^7^F_0_ ground state to ^5^D_0_ level where the emission rates B_10_ (s^−1^) of Eu^3+^ occurred in the 592, 614, 653, and 701 nm peaks, as is shown in Figure 5.

Therefore, the following ratio equations are proposed:

(1) $$\frac{dN_{Tb}^{2}}{dt}=A_{02}N_{Tb}^{0}-A_{21}$$

(2) $$\frac{dN_{Tb}^{1}}{dt}=A_{21}N_{Tb}^{2}-A_{10}N_{Tb}^{1}-WN_{Eu}^{0}N_{Tb}^{1}$$

(3) $$\frac{dN_{Eu}^{1}}{dt}=-B_{10}N_{Eu}^{1}+WN_{Eu}^{0}N_{Tb}^{1}$$

where $N_{Tb}^{2}$, $N_{Tb}^{1}$, and $N_{Tb}^{0}$ (ions/cm^3^) are the Tb^3+^ ion populations in the ^5^D_3_, ^5^D_4_, and ^7^F_6_ energy levels, respectively. $N_{Eu}^{1}$ and $N_{Eu}^{0}$ (ions/cm^3^) are the Eu^3+^ ion populations in the ^5^D_0_ and ^1^F_0_ levels, respectively. For low-excitation pumping, the ground populations are proportional to the nominal concentration, i.e., $N_{Tb}^{0}\approx N_{Tb}$ and $N_{Eu}^{0}\approx N_{Eu}$. In stationary conditions the solutions are:

(4) $$N_{Tb}^{1}=\frac{A_{02}N_{Tb}}{\left(WN_{Eu}+A_{01}\right)}$$

(5) $$N_{Eu}^{1}=\frac{WA_{02}N_{Tb}N_{Eu}}{B_{10}\left(WN_{Eu}+A_{01}\right)}$$

(6) $$N_{Tb}^{2}=\frac{N_{Tb}A_{02}}{A_{21}}$$

**Figure 6:**
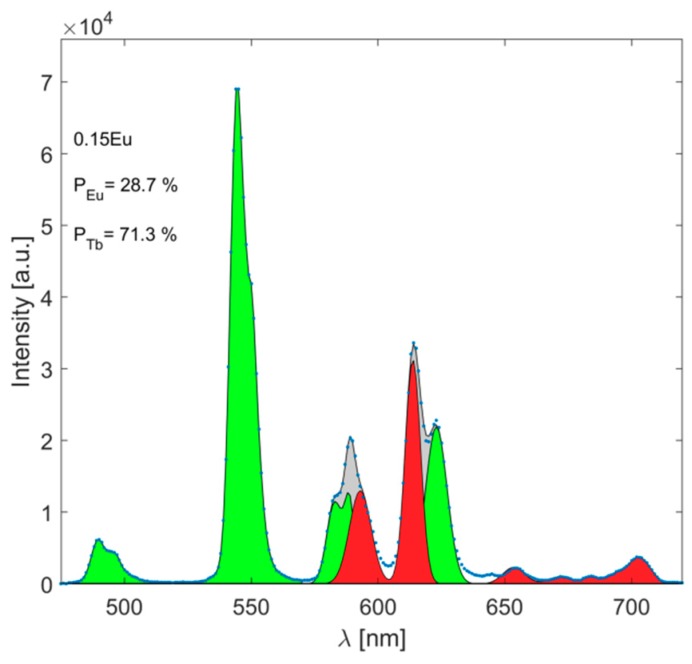
Deconvolution to the experimental emission spectrum (blue). Red and green areas represent the emissions related to the Eu^3+^ and Tb^3+^ ions, respectively.

Then, ion populations are related to the emission spectrum by:

(7) $$N_{Tb}^{1}=k\int^{\text{​}}I_{Tb}d\lambda$$

(8) $$N_{Eu}^{1}=k\int^{\text{​}}I_{Eu}d\lambda$$

where *I_Tb_* and *I_Eu_* are the emission spectra related to the Tb^3+^ and Eu^3+^ emission transitions, and *k* is a proportional constant. Thus, to obtain the *I_Tb_* and *I_Eu_* emission spectra, deconvolution of the spectra was performed for both ions, as is shown in Figure 6. Then, we define:

(9) $$P_{Tb}=\frac{N_{Tb}^{1}}{N_{Tb}^{1}+N_{Eu}^{1}}=\frac{B_{10}}{WN_{Eu}+B_{10}}$$

(10) $$P_{Eu}=\frac{B_{21}N_{Eu}^{1}}{N_{Tb}^{1}+N_{Eu}^{1}}=\frac{WN_{Eu}}{WN_{Eu}+B_{10}}$$

(11) $$P_{Tb}=\frac{\int^{\text{​}}I_{Tb}d\lambda }{\int^{\text{​}}I_{Tb}d\lambda +\int^{\text{​}}I_{Eu}d\lambda }$$

(12) $$P_{Eu}=\frac{\int^{\text{​}}I_{Eu}d\lambda }{\int^{\text{​}}I_{Tb}d\lambda +\int^{\text{​}}I_{Eu}d\lambda }$$

On the other hand, the dynamic solution for Tb^3+^ in the level ^5^D_4_ is:

(13) $$N_{Tb}^{1}\left(t\right)=\frac{A_{21}A_{02}N_{Tb}^{0}}{A_{21}-A_{10}-N_{Eu}^{0}W}\left(e^{-\left(A_{01}+WN_{Eu}^{0}\right)t}-e^{-A_{21}t}\right)$$

This equation has two terms related to the rise time and lifetime. In this way, the lifetime is expressed by:

(14) $$\tau =\frac{1}{A_{01}+WN_{Eu}^{0}}$$

**Figure 7:**
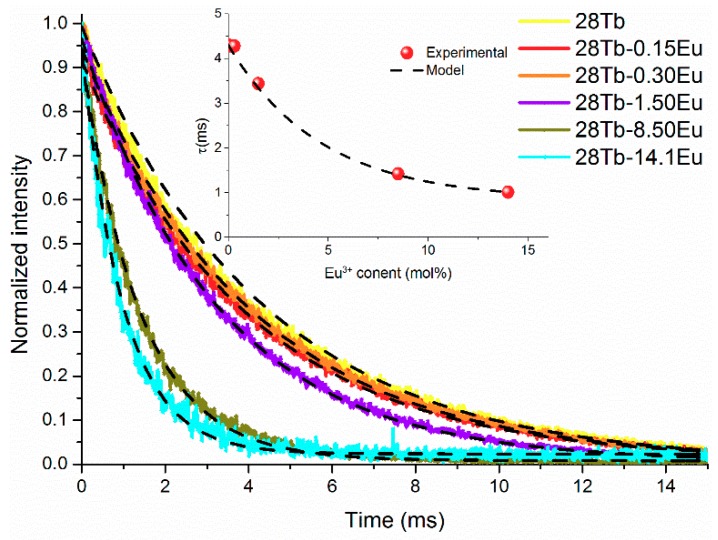
Experimental lifetime curves. The inset is a graph of experimental lifetime. Black lines represent the fitting model.

Figure 7 shows the experimental lifetime curves, and the inset graph is the experimental fitting of Equation (14), with A_01_ = 226.8/s and W = 53.4/s mol%. In our samples, the lifetimes of the ^5^D_4_ level of Tb^3+^ showed values of 4.3, 4.28, 4.28, 3.44, 1.42, and 1.01 ms when Eu^3+^ concentration increased to 0, 0.15, 0.3, 1.5, 8.5, and 14 mol%, respectively (see Table 1). 

**Figure 8:**
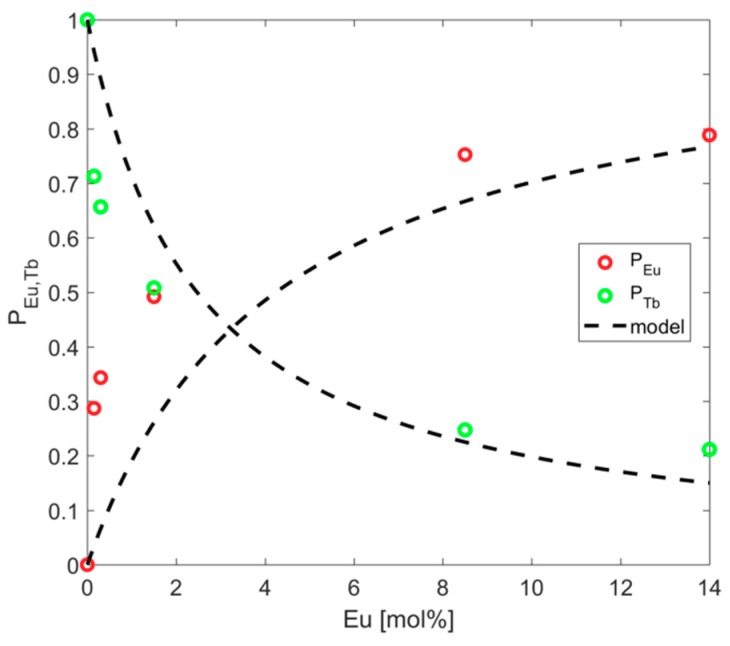
Normalized emission spectrum related to the Eu^3+^ (red) and Tb^3+^ (green) ions. Black lines represent the fitting model.

Figure 8 shows the experimental normalized emission spectra according to Equations (11) and 12. Additionally, adjustment of the experimental data was carried out using Equations (9) and (10), with B_10_ = 131.2/s. The model simultaneously adjusted the emission spectrum and lifetime curves. Luminescent efficiency can be defined by the ratio of population loss by emission and the population gain of the level:

(15) $$\eta =\frac{A_{10}N_{Tb}^{1}}{A_{21}N_{Tb}^{2}}$$

Substituting Equations (4) and (5) we find:

(16) $$\eta =\frac{A_{01}}{A_{01}+WN_{Eu}}$$

Energy transfer efficiency can be defined by the ratio of population loss by energy transfer and the population gain of the level:

(17) $$ET=\frac{WN_{Tb}^{1}N_{Eu}^{0}}{A_{21}N_{Tb}^{2}}=\frac{WN_{Eu}}{A_{01}+WN_{Eu}}$$

Equations (16) and (17) fulfill the following relationship:

(18) ET+η=1

Then, energy transfer can be rewritten as:

(19) $$ET=1-\frac{\tau }{\tau_{0}}$$

where $\tau ={\left(A_{01}+WN_{Eu}\right)}^{-1}$ is the lifetime, and $\tau_{0}={\left(A_{01}\right)}^{-1}$ is the radiative lifetime. In this work, $\tau =\tau_{Tb-Eu}$ and $\tau_{0}=\tau_{Tb}$ were the fluorescence lifetimes of the ^5^D_4_ level of Tb^3+^ for Tb^3+^-doped and Tb^3+^/Eu^3+^-codoped ceramic phosphor plates. The calculated ET efficiency increased from 5 to 76% when the Eu^3+^ concentration increased from 0.15 to 14 mol%. ET increased rapidly to 30% with the addition of 1.5 mol% of Eu^3+^; after this concentration, no big changes were observed, and the ET was kept almost constant at 14 mol% of Eu^3+^.

### 3.3. White Light Device Fabrication

**Figure 9:**
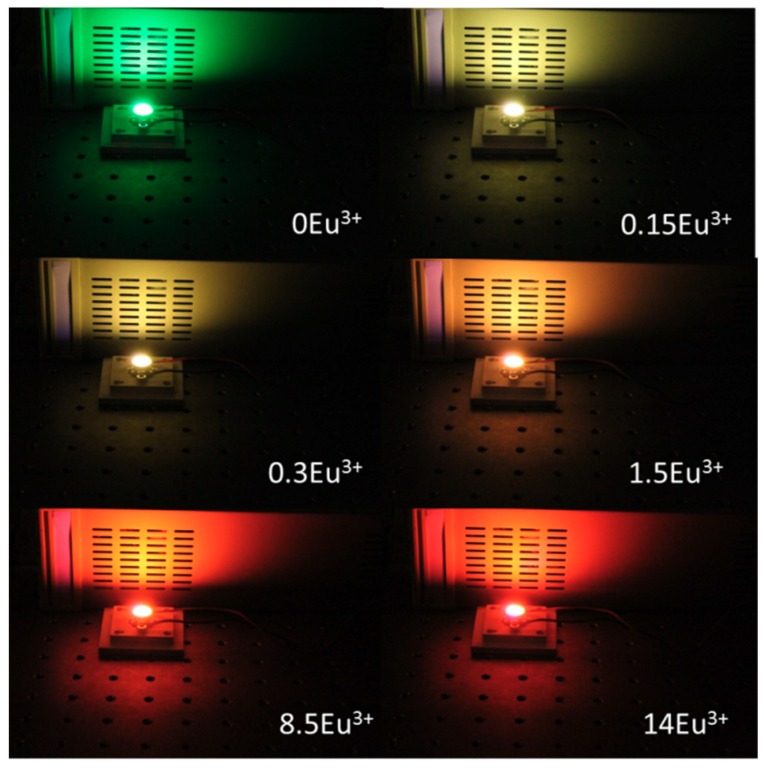
Luminescence photographs of fabricated devices under a 380 nm UV LED chip.

Figure 9 shows multicolor light devices that were constructed using the Tb^3+^/Eu^3+^-codoped ceramic phosphor plate and the 380 nm UV LED chip.

**Figure 10:**
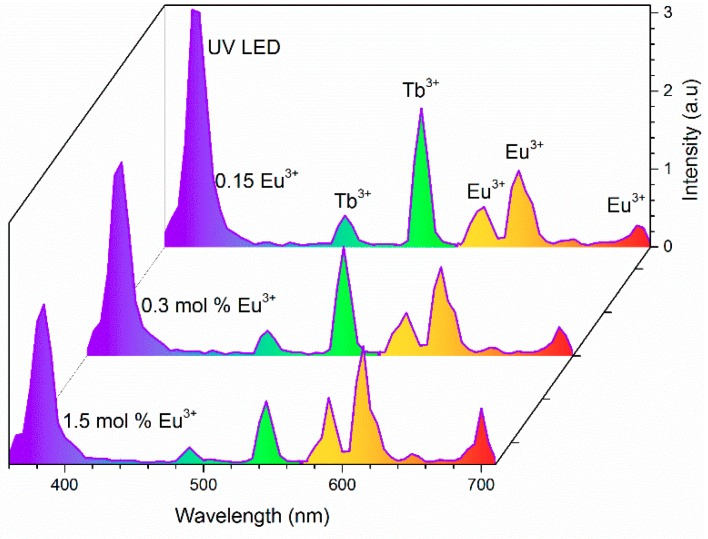
Electroluminescent spectra of LEDs fabricated using a 380 nm UV LED chip combined with Tb^3+^-doped and Tb^3+^/Eu^3+^-codoped ceramic phosphor plates under a forward bias of 20 mA.

The electroluminescence of fabricated devices as a function of Eu^3+^ concentration with a bias current of 20 mA is shown in Figure 10. Representative samples with 0.15, 0.3, and 1.5 mol% of Eu^3+^ clearly showed 380 (UV LED), 544 (Tb^3+^), 592 (Eu^3+^), and 614 nm (Eu^3+^) bands, where warm white was the feature color emissions of these devices. The maximum luminous efficacy was measured to be 13.08 lm/W for 28 mol% of Tb^3+^, whereas a decrement from 9.22 to 6.04 lm/W was observed as the Eu^3+^ concentration increased (see Table 1). The low values of luminous efficacy were associated with the opacity of the samples, the low efficiency of UV LED (InGaN), as well as the poor contribution of color from LED. 

**Figure 11:**
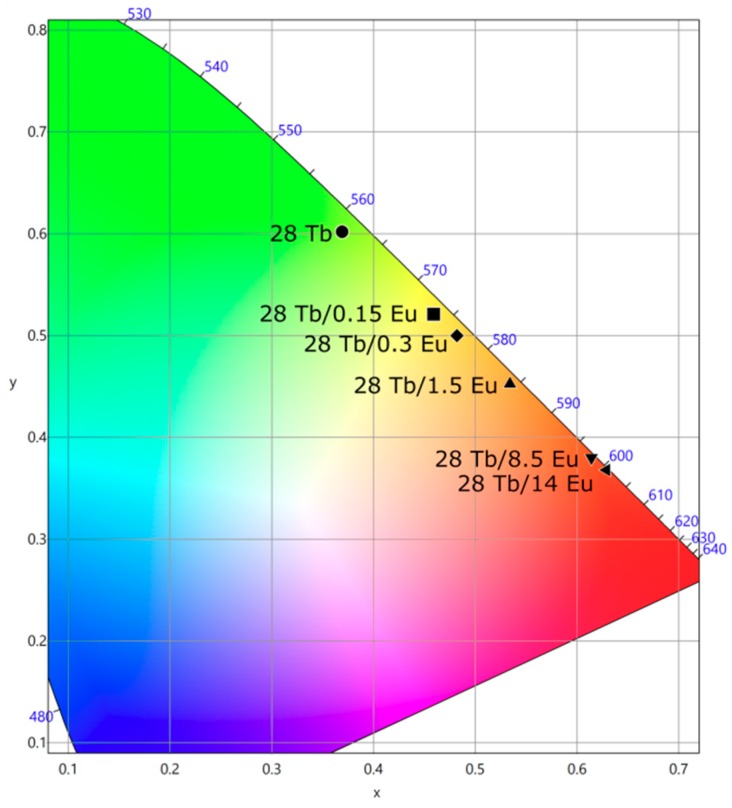
CIE diagram of chromaticity as function of Eu^3+^ content.

Figure 11 shows the color coordinates of the samples under study. The values were located on the edge of the chromaticity diagram, predominantly in yellow and red regions. The emitted color of the device changed from green to red through warm white by keeping the Tb^3+^ concentration and changing the Eu^3+^ content. The obtained results showed that the optical properties of LED were strongly influenced by Eu^3+^ content. Interestingly, by doping with only 0.15 mol% of Eu^3+^, it was possible to modify both CRI and CCT. CRI showed an increase from 34 to 74.81, and CCT diminished from 5497 to 3658 K; these features were very adequate for indoor lighting. The samples with 0.3 and 1.5 mol% of Eu^3+^ gave the highest CRIs of approximately 82.5 and 82.6 with CCTs of 3136 and 2225 K, respectively. Although the luminous efficacy of the devices was low, we expected to further increase such values by introducing Tb^3+^ and Eu^3+^ in an adequate matrix or by codoping with Ce^3+^ to increase the absorption strength. The obtained results showed that by choosing properly the Eu^3+^ concentration, it was possible to produce comfortable white light devices for vivid applications in daily life.

## 4. Conclusions

In summary, the fabrication of LiYF_4_ ceramic phosphor plates doped with Tb^3+^/Eu^3+^ ions is reported. The maximum excitation peak of a single Tb^3+^ was at 373 nm; however, this changed to 378 nm when LiYF^4^:10Tb^3+^ was codoped with Eu^3+^ ions. Based on the experimental results, it was concluded that intensity ratios between emission bands could be tuned by choosing properly the ion concentrations of both Tb^3+^ and Eu^3+^ ions. By placing the LiYF_4_:28Tb^3+^/yEu^3+^ (mol%) ceramic phosphor plates on the top of 380 nm LED chip, green, warm, and red color emissions were obtained. It was found that warm white was achieved by adding only 0.15 mol% of Eu^3+^ without serious detriment to the luminous efficacy. However, when the concentration of Eu^3+^ increased to 0.3 mol% in LiYF_4_:28Tb^3+^/yEu^3+^ (mol%), a CRI of 82.3 and a CCT of 3136 K were measured. With an increase in Eu^3+^ concentration, the yellow and red bands were improved, but the blue and green bands were reduced. Then, it was necessary to compromise CRI, CCT, and luminous efficacy to define the ion concentration. The obtained CRI was one of the highest reported in the literature for the Tb^3+^/Eu^3+^ system, which, in combination with low CCT, made LiYF_4_ ceramic phosphor plates a good candidate for solid-state lighting applications.

## Figures and Tables

**Table 1:** Decay time, energy transfer, color coordinate (CC), color-correlated temperature (CCT), color rendering index (CRI), and luminous efficacy (LE) of the ceramic phosphor plate as a function of Eu^3+^ content.

Tb^3+^/Eu^3+^ (mol%)	τ_Tb_(^5^D_4_) (ms)	ET (%)	x	y	CCT (K)	CRI	LE (lm/W)
28	4.3	0	0.369	0.602	5497	34.06	13.08
28/0.15	4.28	0.4	0.459	0.521	3658	74.81	9.22
28/0.3	4.28	0.4	0.482	0.500	3136	82.54	8.13
28/1.5	3.44	20	0.534	0.453	2225	82.59	7.09
28/8.5	1.42	66	0.614	0.381	1136	42.81	7.80
28/14	0.93	78	0.629	0.368	1294	35.54	6.04
